# Bactericidal activity of Myrrh extracts and two dosage forms against standard bacterial strains and multidrug-resistant clinical isolates with GC/MS profiling

**DOI:** 10.1186/s13568-020-0958-3

**Published:** 2020-01-28

**Authors:** Noha Khalil, Sahar Fikry, Osama Salama

**Affiliations:** 1 Faculty of Pharmaceutical Sciences and Pharmaceutical Industries, Future University in Egypt, Cairo, 11835 Egypt; 2 Faculty of Applied Health Sciences Technology, Pharos University in Alexandria, Alexandria, 21311 Egypt

**Keywords:** Essential oil, *S. aureus*, *Ps. aeruginosa*, Myrrh cream, Myrrh mouthwash, Multi-resistant clinical isolates

## Abstract

Myrrh is the resinous exudate obtained by the incision in *Commiphora molmol* trees (Family Burseraceae). The bactericidal activity of its hexane extract was compared to its essential oil (MEO) using viable count technique against *Staphylococcus aureus* (*S. aureus*) and *Pseudomonas aeruginosa* (*Ps. aeruginosa*). MEO exhibited a better activity with > 99.999% killing of both tested strains after 2 h contact time. MEO was tested using the same technique against four multidrug resistant isolates: *S. aureus* (MRSA, sputum), *Escherichia coli* (*E. coli*, urine), *Ps. aeruginosa* (wound) and *Klebsiella pneumonia* (*K. pneumonia*, sputum). Highest bactericidal activity was observed against *Ps. aeruginosa* while lowest was against *K. pneumonia* (99.59 and 54.04% killing, respectively after 2 h contact time). A cream and mouthwash were formulated using 5% v/v MEO. The cream showed a better activity against *Ps. aeruginosa* than *S. aureus* (95.11 and 86.76% killing, respectively after 2 h contact time). The in vitro treatment of ca 10^7^ CFU/ml *S. aureus* cells suspended in 10% saliva with the mouthwash produced ca 46% killing within the first 15 min reaching ca 99.999% after 30 min. Cytotoxic studies of both the essential oil and hexane extract on human liver cancer (Hep G2), human breast cancer (MCF-7) and colon cancer cell lines (HCT-116) revealed a promising in vitro activity. Highest activity was recorded for the essential oil on MCF-7 with IC_50_ 10.93 ± 0.32 μg/ml. GC/MS analysis allowed the identification of 17 and 9 compounds representing 92.01 and 99.99% of the hexane extract and essential oil, respectively. Furano-eudesma-1,3-diene (15.99%) and 2-acetoxy-furano-diene (26.82%) were the major identified compounds in the hexane extract and essential oil, respectively. These results indicate that Myrrh essential oil is a promising antibacterial and cytotoxic agent that can be formulated in suitable dosage forms.

## Introduction

Myrrh, as a popular traditional natural medicine, is a yellowish oleo-gum resin obtained as an exudate from the stems and branches of *Commiphora molmol* (Nees) Engl. and other species of *Commiphora* belonging to family Burseraceae (Tucker 1986). It is mainly produced in China, India and Middle East. Chemically, Myrrh is composed of essential oil, water-soluble gum and alcohol-soluble resin (El Ashry et al. 2003). Myrrh has been widely employed as a common analgesic and in cleaning wounds and sores for more than 2000 years, until the discovery of morphine (Dolara et al. 1996). Myrrh is one of the oldest known medicines which have been widely used by ancient Egyptians (El Ashry et al. 2003). Myrrh is famous in the Chinese traditional medicine along with the resinous Frankincense for the treatment of blood stagnation (known together as the blood moving medicine), inflammatory diseases as well as relief of swelling and pain (Fatani et al. 2016; Shen and Lou 2008). Recently, Myrrh has been reported to have antiseptic, analgesic and antipyretic activities (Shalaby and Hammouda 2014). Furthermore, studies show that it can be used as an antirheumatic (Su et al. 2015), antiparasitic (El-Sayad et al. 2017), hypotensive (Abdul-Ghani and Amin 1997) and hypolipidemic agent (Omer and Al-Dogmi 2018). A cytotoxic activity against MCF-7 and HS-1 cells was also reported (Chen et al. 2013). Several studies reported the antimicrobial activity of Myrrh and its extracts against different types of pathogens (Mahboubi and Mohammad 2016; Shuaib et al. 2013). *C. molmol* essential oil has proven a strong antifungal activity especially against *Aspergillus flavus, Cladosporium* sp., *Aspergillus alternata*, *Fusarium oxysporum* and *Fusarium solani* (Perveen et al. 2018). Moreover, several isolated compounds from Myrrh have proved good antibacterial activity. Methoxyfuranoguaia-9-ene-8-one and furanodiene-6-one have shown potent growth inhibition activity, with minimum inhibitory concentrations (MIC) values ranging between 0.2 and 2.8 µg/ml (Dolara et al. 2000). Similarly, sesquiterpenoids like β-elemene and T-cadinol isolated from Myrrh oleo-resin inhibited the growth of different pathogenic bacteria, with MIC ranging between 4 and 256 µg/ml (Rahman et al. 2008). The present study aimed to evaluate the bactericidal activity of Myrrh essential oil as well as its hexane extract against standard microorganisms and multi-drug resistant clinical isolates using viable count technique, as well as to evaluate the change in bactericidal activity after formulating the essential oil into a cream and mouthwash. Cytotoxic studies of the essential oil as well as the hexane extract was tested on normal hamster lung fibroblasts (V79 cells), human liver cancer (Hep G2), human breast cancer (MCF-7) and colon cancer cell lines (HCT-116). Moreover, chemical composition of both the essential oil and hexane extract was achieved through GC/MS analysis.

## Materials and methods

### Plant material

Oleo gum resin of *C. molmol* was obtained from Gattefossé SAS Co., France. The oleo gum resin was grounded into coarse powder by a grinder. Voucher specimen was kept at the Faculty of Pharmaceutical Sciences and Pharmaceutical Industries, Future University in Egypt (labeled M-12).

### Preparation of the hexane extract

Myrrh powder (500 mg) was extracted using hexane by maceration for 24 h at room temperature with occasional shaking (250 g × 2 L). The extracts were then filtered and dried by rotary evaporation.

### Extraction of the essential oil

Myrrh powder was subjected to hydro-distillation for 5 h using a Clevenger apparatus. Obtained essential oil was dried over anhydrous sodium sulfate. The oil was refrigerated at 4 ^°^C in a sealed amber vial till use.

### Source of microorganisms and culture media

Three standard laboratory reference strains from American Type Collection Culture (ATCC) for bacteria (purchased from IMTECH, Chandigarh, India) were used for determination of the bactericidal activity. The tested microorganisms are listed in Table 2. Cultures were adjusted to 0.5 McFarland standard which contains approximately 1 to 2 × 10^8^ CFU/ml with tested bacterial strains, then dilute the 0.5 McFarland suspension 1:10 in sterile broth or saline to obtain a concentration of 10^7^ CFU/ml, the adjusted suspensions for final inoculation should be used within 15 min of preparation.

### Clinical isolates

Four multi-resistant clinical isolates (Table 3) were obtained from Department of Pharmaceutical Microbiology, Faculty of Pharmacy, University of Alexandria, and their identity was ascertained by classical procedures (Berkowitz and Jerris 2015).

### Determination of bactericidal activity by viable count technique

Stable stock emulsion of the oil was prepared using Cremophor El (triturating ten volumes of the oil with one volume of Cremophor El (10 ml oil + 1 ml Cremophor El) (Wisher 2012). The resultant emulsion was sterilized by filtration though 0.45 µm membranes filter (Millipore, USA). Aliquots of each emulsion were properly diluted with sterile water, inoculated with overnight culture of test organism diluted with water (1:100), vortexed and incubated at 37 °C. The same volume of Cremophor El completed to 100 ml with water was used as a negative control. At specified time intervals, the inoculated systems were vortexed, and aliquots were decimally diluted with sterile saline and the number of viable cells was determined by transferring 20 µl portions of each dilution onto the surface of overdried soybean casein Digest agar (Oxoid) plates (Sigma, USA). These were incubated at 37 °C for 48 h and the number of developed colonies was counted and average number of cells calculated as CFU/ml. Controls lacking tested products were included in the test. For determination of the effect of MEO emulsion concentration, the same procedure was followed and oil concentration varied between 0 and 5% v/v.

### Determination of antibiotic resistance

Antibiotic resistance pattern of clinical isolates was determined by single disk agar diffusion technique using 26 different Oxoid-made antibiotic susceptibility disks and Muller Hinton agar (Oxoid). The resultant inhibition zones were translated into antibiotic resistance pattern using published tables.

### Preparation of the cream and determination of its bactericidal activity

The cream was made up of the following [g%]: MEO emulsion [5], stearic acid [13], stearyl alcohol [1.0], cetyl alcohol [1.0], potassium hydroxide [1], glycerol [10] and distilled water [Q.S to 100].

One gram quantities of the prepared cream were distributed into sterile small beakers. Each beaker then received 100 µl of an overnight culture of the organism and mixed thoroughly using sterile glass rods under laminar air flow cabinet. At the specified times, 9 ml of sterile water were added to each inoculated cream, mixed well, decimally diluted with sterile water and the number of viable cells was determined by surface viable count technique. As a control a placebo cream was included in the test.

### Preparation of the mouthwash and determination of its bactericidal activity

Mouthwash was made up of the following [g%]: MEO emulsion [5], cremophor El [2.5], lidocaine HCl [0.01], citric acid [0.03], sodium citrate [0.07], saccharin sodium [0.02] and distilled water [Q.S. to 100 ml].

10 ml of mouthwash were decimally diluted with sterile saline and used for the determination of the number of surviving organisms using the described surface viable count method. The test was repeated using placebo mouthwash as control. The percent recovered of microbial cells (CFU/ml) was plotted against time following application of mouthwash. Listerine^®^ was used as a standard mouthwash for comparison.

### In-vitro cytotoxic activity

#### Human tumor cell lines

Hep G2 (human liver cancer), HCT-116 (human colon carcinoma) cell lines and MCF-7 (human breast adenocarcinoma) cells, maintained in the laboratory of Cancer Biology Department of National Cancer Institute in Egypt, were used for in vitro cytotoxicity assay. A control was included using normal hamster lung fibroblasts (V79 cells).

#### Cytotoxicity assay

Different concentrations of the essential oil and hexane extract (0–200 µg/ml) were tested for cytotoxicity against the selected human cancer cell lines using sulforhodamine B stain (SRB) method (Skehan et al. 1990). The relation between survivals and the oil concentration was plotted to get the survival curve of each tumor cell line after the application of specific concentration. The results were compared to those of the standard cytotoxic drug, doxorubicin (10 mg adriamycin hydrochloride, in 5 ml IV injection, Pharmacia, Italy) at the same concentrations were used as standard cytotoxic agent. The dose of the test solutions which reduced survivals to 50% (IC_50_, µg/ml) was calculated together with selectivity index.

### Gas chromatography/mass spectrometry (GC/MS) analysis of the essential oil and hexane extract

An Agilent 7890A gas chromatograph (Agilent Technologies, Palo Alto, CA, USA) with a capillary column RTX-5MS (30 m × 0.32 mm, film thickness 0.25 μm) was used for the GC/MS analysis of the essential oils. This was coupled to an Agilent 5975C mass selective detector. The initial oven temperature was 40 °C for 2 min, then it was raised at the rate of 5 °C/min until it reached 210°. The injector and detector temperatures were 290 and 300 °C, respectively. Helium carrier gas was used at a flow rate of 2 ml/min. Manual split mode injection was applied (0.1 μl, each). EI mode was used for recording the mass spectra. The range for m/z was 35–500. Ionization voltage was 70 eV and ion source temperature was set at 230 °C. The above conditions were applied for the analysis of a homologous series of n-alkanes to calculate Kovat’s index (KI). Identification was based on comparison of KI with literature (Adams 2007), in addition to obtained data from Wiley’s MS libraries. Authentic compounds (Sigma-Aldrich, Germany) were also used for identification of some compounds.

### Gas chromatography/flame ionization detection (GC-FID)

The GC analyses were carried out on a Focus GC^®^ (Thermo fisher scientific^®^, Milan, Italy) equipped with TR5-MS fused bonded column (30 m × 0.25 mm × 0.25 µm) (Thermo fisher scientific^®^, Florida, USA) and FID detector; carrier gas was nitrogen (1.5 ml/min); the operating conditions were: initial temperature 40 °C, 1 min isothermal followed by linear temperature increase till 230 °C at a rate of 4 °C/min, then 5 min isothermal. Detector and injector temperatures were 300 and 220 °C, respectively. The split ratio was 1: 20. Chrom-card^®^ chromatography data system ver. 2.3.3 (Thermo Electron Corp. ^®^, Florida, USA) was used for recording and integrating of the chromatograms. Average areas under the peaks of three independent chromatographic runs were used for calculation the % composition of each component.

### Statistical analysis

Tests were conducted in triplicate and values recorded as mean ± SEM. Results were analyzed by GraphPad Prism^®^ v.5 software. Significant differences among means of different samples were analyzed using paired-*t*-test at p ≤ 0.05 for all analysis except cytotoxic analysis which was separated using Bonferroni posttests at p ≤ 0.05.

## Results

### Bactericidal activity of Myrrh essential oil (MEO) relative to the hexane extract

Hexane extract of Myrrh yielded 33.5 ± 2.5% w/w and hydro-distillation yielded 6.5 ± 0.5% v/v essential oil. In a preliminary experiment, *Staphylococcus aureus* and *Ps. aeruginosa* cells (ca 10^7^ CFU/ml) were exposed for 5 min to the water soluble components of MEO (5% v/v) obtained by intermittent shaking of the oil with water. The survived cells were about 4% and < 0.001% for the two tested organisms, respectively (data not shown).

However, for better solubilization and formulation, stable emulsions of MEO and hexane extract were prepared in Cremophor El and used in all the experiments. The bactericidal activity of MEO emulsion was compared to the hexane extract emulsion against *S. aureus* and *Ps. aeruginosa* using viable count technique (Table 1). Generally, *Ps. aeruginosa* was relatively more sensitive to the tested emulsions than *S. aureus* particularly during the first 30 min of contact. MEO exhibited a better activity with > 99.999% killing of both tested strains after 2 h contact time. Consequently, the MEO emulsion was used in the remaining part of the work.

**Table 1:** Bactericidal activity of hexane extract and essential oil of Myrrh tested by surface viable count method on standard microorganisms

Test organism	Contact time (h)	Hexane extract (5%)	Essential oil (5%)
		Viable count, CFU/ml^a^ (% killing)	
*S. aureus* (ATCC 6538)	0	5.0 × 10^7^	8.5 × 10^7^
	0.5	4.8 × 10^7^ (40.00)	6.8 × 10^7^ (20.00)
	2	3.0 × 10^7^ (40.00)	2.0 × 10^1^ (> 99.999)
*Ps. aeruginosa* (ATCC 9027)	0	9.25 × 10^7^	5.0 × 10^7^
	0.5	1.83 × 10^7^ (98.03)	1.95 × 10^6^ (96.10)
	2	1.7 × 10^3^ (> 99.998)	2.0 × 10^1^ (> 99.999)

^a^Average of three determinations carried out by surface viable count method at RT (24 °C)

### Bactericidal activity of different concentrations of MEO

The bactericidal activity of different concentrations of MEO emulsion (1, 2, 5% v/v) were tested against three standard strains (*S. aureus, E. coli* and *Ps. aeruginosa*) using viable count technique (Table 2). *Ps. aeruginosa* was the most sensitive organism while *E. coli* was the least affected. This was quite evident from the effect of 1% MEO which in 2 h killed > 99.999%, 99.36% and 44.25% of the exposed cells of the three tested bacteria, respectively. More than 99.999% of *Ps. aeruginosa* and *S. aureus* were killed after 2 h exposure to the 5% v/v MEO. This concentration (5% v/v) was therefore chosen to test against multidrug resistant clinical isolates as well as formulation of the cream and mouthwash.

**Table 2:** Bactericidal activity of different concentrations of the essential oil of Myrrh against three standard bacterial strains tested by surface viable count method

Test organism	Contact time (h)	Essential oil (%)			
		0	1	2	5
		Viable count, CFU/ml^a^ (% killing)			
*S. aureus* (ATCC 6538)	0.0	8.5 × 10^7^	8.5 × 10^7^	8.5 × 10^7^	8.5 × 10^7^
	0.5	–	8.0 × 10^7^ (6.88)	6.8 × 10^7^ (20.00)	5.7 × 10^7^ (32.94)
	2.0	–	5.4 × 10^5^ (99.36)	2.47 × × 10^5^ (99.71)	2.0 × 10^1^ (> 99.999)
	24.0	3.3 × 10^8^	< 2.0 × 10^1^ (> 99.999)	< 2.0 × 10^1^ (> 99.999)	< 2.0 × 10^1^ (> 99.999)
*E. coli* (ATCC 8739)	0.0	8.0 × 10^7^	8.0 × 10^7^	8.0 × 10^7^	8.0 × 10^7^
	2.0	–	4.3 × 10^7^ (44.25)	3.1 × 10^7^ (73.75)	1.95 × 10^7^ (75.61)
	24.0	1.8 × 10^8^	2.7 × 10^6^ (98.50)	3.0 × 10^4^ (99.983)	> 2.0 × 10^1^ (> 99.999)
*Ps. aeruginosa* (ATCC 9027)	0.0	5.0 × 10^7^	5.0 × 10^7^	5.0 × 10^7^	5.0 × 10^7^
	0.5	–	3.7 × 10^7^ (30.00)	2.85 × 10^7^ (43.00)	2.95 × 10^6^ (94.10)
	2.0	–	< 2.0 × 10^1^ (> 99.999)	< 2.0 × 10^1^ (> 99.999)	< 2.0 × 10^1^ (> 99.999)

^a^Average of three determinations carried out by surface viable count method at RT (24 °C)

### Bactericidal activity of MEO against multidrug resistant clinical isolates

Four clinical isolates (*S. aureus* from sputum, *E. coli* from urine, *Ps. aeruginosa* from wound and *K. pneumonia* from sputum) were selected to test the bactericidal activity of MEO emulsion (5% v/v). The isolates demonstrated multidrug resistance as shown in the antibiotic resistance pattern in Table 3. The isolates were resistant to 14–21 of the 26 tested antibiotics, including amoxicillin/clavulanate, piperacillin, third generation cephalosporin’s, chloramphenicol, fluoroquinolones and tetracycline.

**Table 3:** Antibiotic resistance pattern of four clinical isolates

Isolate code (source)	Antibiotic resistance pattern
*S. aureus* (MRSA), S4 (sputum)	AMC, AMP, AK, B, C, CAZ, CE, CEC, CFP, CFR, CIP, CN, CRO, CXT, E, KF, ME, OFX, OT, PRL, SXT
*E. coli,* Eg (urine)	AMC, AMP, C, CAZ, CE, CEC, CFP, CER, CIP, CN, CRO, CXT, KF, PRL. OFX, OT, SXT
*Ps. aeruginosa,* P_2_ (wound)	AMC, AMP, AK, C, CE, CEC, CAZ, CFP, CFR, CIP, CN, CRO, CXT, OFX, PRL, OT, SXT
*K. pneumonia,* KCI (sputum)	AMC AMP, C, CAZ, CE, CEC, CFP, CIP, CNI, CTX, OFX, OT, PRL, SXT

MRSA: methicillin-resistant *Staphylococcus aureus*, AK; amikacin AMC: amoxicillin/clavulanate; AMP: ampicillin; B: bacitracin; C: chloramphenicol; CAZ: ceftazidime; CE: cephradine; CEC: cefaclor; CFP: cefoperazone; CFR: cefadroxil; CIP: ciprofloxacin; CN: gentamicin; CRO: ceftriaxone; CTX: cefotaxime; E: erythromycin; KF: cephalothine; ME: methicillin; OFX: ofloxacin; OT: oxytetracycline; PRL: piperacillin; SXT: sulfamethoxazole/trimethoprim

Exposure of ca 5 × 10^−7^–1 × 10^8^ CFU/ml of MRSA, *E. coli*, *Ps. aeruginosa* and *K. pneumonia* to 5% v/v MEO emulsion for 2 h resulted in 54–99.59% killing. Although the first three isolates gave comparable response, *Ps. aeruginosa* was the highest, on the other hand the percentage of *K. pneumonia* killing was markedly the lowest (Table 4).

**Table 4:** Bactericidal activity of the essential oil of Myrrh (5% v/v) against four multidrug resistant clinical isolates

Isolate code (source)	Contact time (h)	Viable count (CFU/ml)*	Bactericidal activity (% killing)
*S. aureus* (MRSA) S4 (sputum)	0.0	6.00 × 10^7^	–
	0.5	3.50 × 10^7^	41.67
	2.0	2.55 × 10^6^	95.75
*E. coli,* Eg (urine)	0.0	5.00 × 10^7^	–
	0.5	3.50 × 10^7^	30.00
	2.0	1.40 × 10^6^	97.20
*Ps. aeruginosa*, P2 (wound)	0.0	1.15 × 10^8^	–
	0.5	4.85 × 10^7^	57.83
	2.0	4.70 × 10^5^	99.59
*K. pneumonia,* KCl (sputum)	0.0	8.05 × 10^7^	–
	0.5	3.90 × 10^7^	51.55
	2.0	3.70 × 10^7^	54.04

* CFU/ml obtained from average of three determinations carried out by surface viable count method at RT (24 °C)

### Bactericidal activity of MEO cream

A cream was formulated using MEO emulsion (5% v/v). Its bactericidal activity was tested against two standard strains; *S. aureus* and *Ps. aeruginosa* using viable count technique. The bactericidal activity of the formulated cream varied among the two test organisms (Table 5). Response against *Ps*. *aeruginosa* was considerably higher than that against *S. aureus* as evidenced from the percentage killing values which were 95.11% and 86.76%, respectively after 2 h exposure.

**Table 5:** Bactericidal activity of Myrrh essential oil cream (5% v/v)

Test organism	Contact time (h)	Viable count (CFU/ml)	Bactericidal activity (% killing)
*S. aureus* (ATCC 6538)	0.0	1.70 × 10^7^	–
	0.5	1.05 × 10^7^	38.24%
	2.0	2.25 × 10^6^	86.76
*Ps. aeruginosa* (ATCC 9027)	0.0	2.25 × 10^7^	–
	0.5	1.05 × 10^7^	53.33%
	2.0	1.10 × 10^6^	95.11

Average of three determinations carried out by surface viable count method at RT (24 °C)

### In-vitro bactericidal activity of MEO mouthwash

**Fig. 1:**
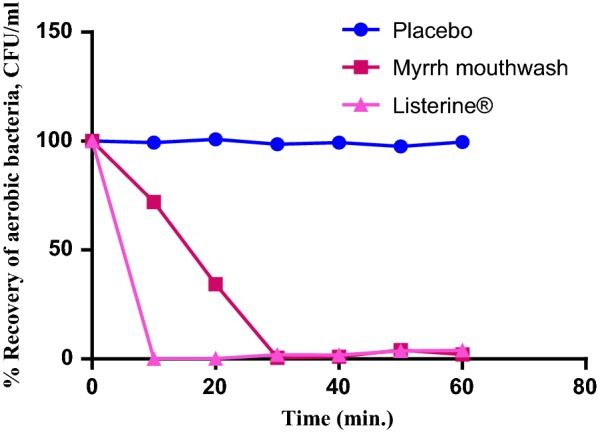
In vitro antibacterial activity of Myrrh mouthwash (5% v/v) compared to placebo and Listerine^®^

A mouthwash was formulated using MEO emulsion (5% v/v) and its bactericidal activity was tested in vitro against *S. aureus* using viable count technique (Fig. 1). The in vitro treatment of ca 10^7^ CFU/ml *S. aureus* cells suspended in 10% saliva with the mouthwash produced ca 46% killing within the first 15 min reaching ca 99.999% after 30 min and remained so for next 30 min (till the rest of the experiment). The activity was close to that of Listerine^®^, except that the latter responded faster.

### In-vitro cytotoxicity of Myrrh hexane extract and essential oil

**Fig. 2:**
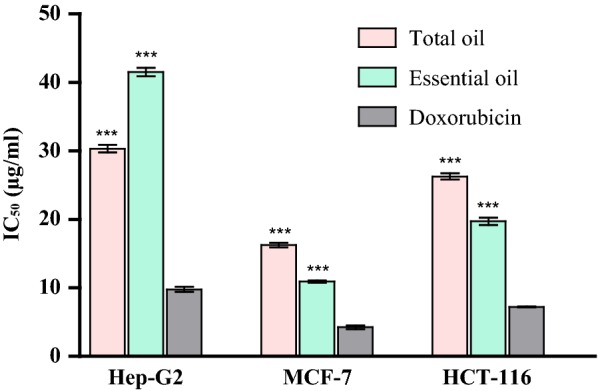
In-vitro cytotoxic activity of Myrrh hexane extract and essential oil on the tested cell lines. Significant differences among means of different treatments were separated using Bonferroni posttests at p ≤ 0.05 (n = 3) with all treatments compared to the control; doxorubicin. ***p < 0.001

In-vitro testing of cytotoxic activity of Myrrh hexane extract and essential oil using SRB assay revealed a strong cytotoxic activity (IC_50_ < 20 μg/ml) for the essential oil and hexane extract on MCF-7 cell lines (IC_50_; 16.32 ± 0.65 and 10.93 ± 0.32 µg/ml, respectively) and for the essential oil on HCT-116 cell lines (IC_50_; 19.71 ± 0.92 µg/ml). However, both the essential oil and hexane extract showed less activity on the other tested cell lines with IC_50_ ranging between 26.28 ± 0.78 and 41.52 ± 1.11 µg/ml. Generally, the essential oil exhibited better cytotoxic activity than the hexane extract, except on liver cancer cell lines where the hexane extract had better activity with lower IC_50_ value (Table 6, Fig. 2).

**Table 6:** In-vitro cytotoxic activity of Myrrh hexane extract and essential oil

Test	Liver cancer cell line (Hep G2)	Selectivity index (SI)	Breast tumor cell line (MCF-7)	Selectivity index	Colon cancer cell line (HCT-116)	Selectivity index (SI)
	IC_50_ (μg/ml)		IC_50_ (μg/ml)		IC_50_ (μg/ml)	
Hexane extract	30.33 ± 0.99^aB^	3.50	16.32 ± 0.65^aB^	3.66	26.28 ± 0.78^aB^	3.25
Essential oil	41.52 ± 1.11^bB^	2.85	10.93 ± 0.32^bB^	3.47	19.71 ± 0.92^bB^	3.69
Doxorubicin	9.79 ± 0.62^A^		4.25 ± 0.44^A^		7.22 ± 0.17^A^	

Values are ± SEM (n = 3), means followed by different letters in same column denote significant difference at p < 0.05, paired-*t*-test. Lowercase letters compare means of Myrrh hexane extract and essential oil, uppercase letters compare means of sample with the standard doxorubicin

Selectivity index was calculated as the ratio of the IC_50_ values on normal hamster lung fibroblasts (V79) to those in the tested cancer cell lines. SI > 3 indicates a promising activity

### GC/MS analysis of MEO

**Fig. 3:**
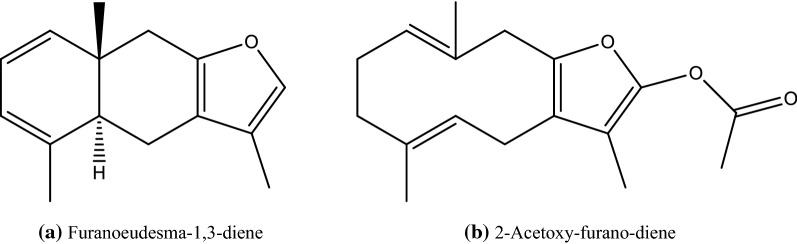
Major identified compound in Myrrh. **a** Hexane extract, **b** essential oil

GC/MS analysis of Myrrh oils allowed the identification of 17 and 9 compounds representing 92.01 and 99.99% of the hexane extract and essential oil, respectively. Furan-type terpenoids prevailed in both the essential oil and hexane extract (Table 7; Fig. 3; Additional file 1: Figures S1, S2). Major identified compounds in the hexane extract included furano-eudesma-1,3-diene (15.99%), isofuranogermacrene (14.26%), furanodiene (10.29%) and 2-acetoxyfuranodiene (9.21%). Compounds identified in the essential oil included 2-acetoxy-furano-diene (26.82%,) furano-eudesma-1,3-diene (20.4%), furano-eudesma-1,4-diene-6-one (17.59%) and isofuranogermacrene (13.52%). Eight compounds present in the hexane extract were completely absent from the essential oil fraction.

**Table 7:** Identified constituents in hexane extract and essential oil of Myrrh

No.	Compound	KI*	Rel. abundance (%)		Methods of identification
			Hexane extract	Essential oil	
*Oxygenated monoterpenes*					
1	Eugenol	1532	3.15 ± 0.32	–	KI, MS, AT
2	Cuminaldehyde	1478	0.71 ± 0.05	–	KI, MS, AT
			3.86		
*Sesquiterpene hydrocarbons*					
3	γ-elemene	1420	0.95 ± 0.12	–	KI, MS, AT
4	δ-Cadinene	1460	4.29 ± 0.21^a^	4.20 ± 0.32^a^	KI, MS, AT
			5.24	4.20	
*Oxygenated sesquiterpenes*					
5	α-copaene-8-ol	1383	1.42 ± 0.12^a^	0.47 ± 0.09^b^	KI, MS, AT
6	Furanodiene	1498	10.29 ± 0.75^a^	4.60 ± 0.24^b^	KI, MS
7	Furanoeudesma-1,3-diene	1518	15.99 ± 0.88^a^	20.40 ± 2.01^b^	KI, MS
8	Lindestrene	1520	5.69 ± 0.95^a^	3.60 ± 0.85^a^	KI, MS
9	Isofuranogermacrene	1452	14.26 ± 0.72^a^	13.52 ± 1.62^a^	KI, MS
10	2-methoxyfuranodiene	1548	3.87 ± 0.33	–	KI, MS
11	2-methoxyfurano-guaia-9-en-8-ne	1572	3.15 ± 0.74	–	KI, MS
12	2-acetoxy-furano-diene	1605	9.21 ± 1.23^a^	26.82 ± 2.08^b^	KI, MS
13	Guaiol	1455	1.67 ± 0.78^a^	8.79 ± 0.68^b^	KI, MS
14	Epi-curzerenone	1510	0.56 ± 0.06	–	KI, MS, AT
15	Furanoeudesma-1,4-dien-6-one	1518	7.96 ± 0.46^a^	17.59 ± 2.79^b^	KI, MS
16	Furanodiene-6-one	1620	6.12 ± 1.66	–	KI, MS
17	4,5-dihydrofuranodiene-6-one	1560	2.72 ± 0.29	–	KI, MS
			82.91	95.79	
	Total % of identified compounds		92.01	99.99	
	Total number of identified compounds		17	9	

MS: Identification based on mass spectral data; AT: Identification based on co-chromatography with authentic samples, (–) = not detected

Values are ± SEM (n = 3), means followed by different letters in same row denote significant difference at p < 0.05, paired-*t*-test

* KI: Kovat’s index calculated using homologous series of *n*-alkanes

## Discussion

Multidrug pharmaceutical preparations containing Myrrh oil are commercially available; in addition, Mirazid^®^, an anthelmintic soft gel capsules containing purified Myrrh oleo-resin extract. Myrrh hexane extract and essential oil are hydrophobic products of *C. molmol.* For formulation of stable liquid forms, they should be solubilized or emulsified. In the present work, the water soluble components of MEO were highly bactericidal against *Ps. aeruginosa* and to a lesser extent *S. aureus.* Cremophor El, a polyoxyl 35 castor oil, was used as an effective emulsifier; however, it reduced the bactericidal activity of MEO. This was evident from the following data; the water-soluble components of MEO in water in 5 min exerted 86% and > 99.999% killing of *S. aureus* and *Ps. aeruginosa* cells, respectively. On the other hand 5% MEO emulsified with Cremophor El provided 20 and 96% killing of the two organisms respectively at the end of 30 min exposure. Similar reductions in the antimicrobial activities of essential oils has been reported in the presence of polysorbates (Nielsen et al. 2016). Myrrh hexane extract and essential oils produced somewhat comparable bactericidal activity against *Ps. aeruginosa* and to lower extant *S. aureus*, denoting shared active principle(s). The essential oil of *C. molmol* and related species has been reported to contain a number of constituents, notable among which were furan-type terpenoids and sesquiterpenes (Hanuš et al. 2005). Terpenoids were shown to possess an array of biological activities including molluscicidal (Borkosky et al. 2009), anti-hyperglycemic (Agwaya et al. 2016), local anesthetic (Tsuchiya 2017), cytotoxic (Shoaib et al. 2017) and antimicrobial (Zengin and Baysal 2014). In this study, MEO exhibited a significant bactericidal activity against certain standard Gram-positive and Gram-negative bacteria and against four clinical isolates which were resistant to 14–21 antibiotics including broad spectrum cephalosporins, amoxicillin/clavulanic acid, chloramphenicol, tetracycline and fluoroquinolones. The tested isolates are known to cause variety of respiratory tract, urinary tract, skin and mucous membrane infections (Alanazi et al. 2018; Freystätter et al. 2018). They rapidly develop resistance against many antibiotics via different mechanisms (Kidd et al. 2017; Zhao et al. 2018). Thus, the observed bactericidal activity of MEO made it potentially advantageous particularly since development of microbial resistance against essential oils has not been reported. MEO cream (5%) was prepared and evaluated microbiologically. It produced significant bactericidal activity against *Ps. aeruginosa* and to lower extent, *S. aureus*. This reduction in activity was probably due to fatty base and the presence of alcohols in the cream, which could make hydrophobic ingredients of the essential oil less available for action on the bacterial cells. However, this MEO cream could still be a valuable adjunct to therapy of skin infection, especially those due to Methicillin-resistant *S. aureus* (MRSA) and *Ps. aeruginosa* multidrug resistant strains. One of the oldest applications of Myrrh was as aromatic stimulant in mouthwashes. Myrrh was also shown to be useful for treatment of sore throat, bleeding gums and chronic pharyngitis (Tonkal and Morsy 2008).

In-vitro testing of cytotoxic activity of Myrrh hexane extract and essential oil using SRB assay revealed a strong cytotoxic for both the essential oil and hexane extract on MCF-7 cell lines and for the essential oil on HCT-116 cell lines. The oils also demonstrated a good selectivity index (SI > 3). Previous studies reported that Myrrh could induce apoptosis in several types of cancer (gynecological, lung, pancreas, liver and prostate) (Chen et al. 2013; Cao et al. 2019). GC/MS analysis of Myrrh oils allowed the identification of 17 and 9 compounds representing 92.01 and 99.99% of the hexane extract and essential oil, respectively. Oxygenated monoterpenes represented by eugenol and cuminaldehyde were found only in the hexane extract. Furanosesquiterpenoids prevailed in both the essential oil and hexane extract. Major identified compounds were furano-eudesma-1,3-diene (15.99%) in the hexane extract and 2-acetoxy-furano-diene (26.82%,) in the essential oil. Furanosesquiterpenoids were the major reported class of compounds in *Commiphora* species (Hanuš et al. 2005; Zhu et al. 2001).

The observed in vitro antibacterial activity of MEO mouthwash together with reported anti-inflammatory, antiulcer and astringent effect of this essential oil, give to this mouthwash a promising efficacy in management of oral infections. In conclusion, in vitro bactericidal activity of Myrrh essential oil, reported in the present work, together with the reported beneficial effects of Myrrh products and its good selectivity index (SI > 3) make this oil a promising safe candidate for inclusion in pharmaceutical products favoring the use of natural medicinal products.

## Supplementary information

**Additional file 1: Figure S1.** GC/MS chromatogram of Myrrh hexane extract. **Figure S2.** GC/MS chromatogram of Myrrh essential oil.

## Data Availability

The used strains are available upon request. All obtained data have been included into the manuscript.
