# Supplementary figures and images for: Bactericidal activity of Myrrh extracts and two dosage forms against standard bacterial strains and multidrug-resistant clinical isolates with GC/MS profiling
Source: AMB Express. 2020 Jan 28;10:21. doi: 10.1186/s13568-020-0958-3 (PMC6987268; doi:10.1186/s13568-020-0958-3)

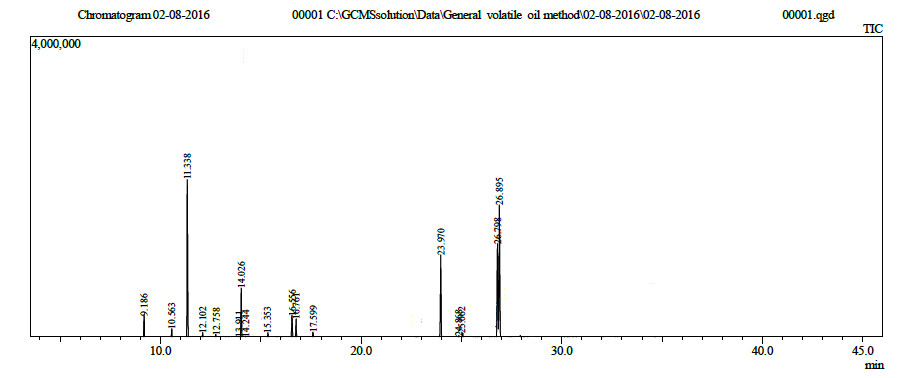


Fig S1: GC/MS chromatogram of Myrrh hexane extract


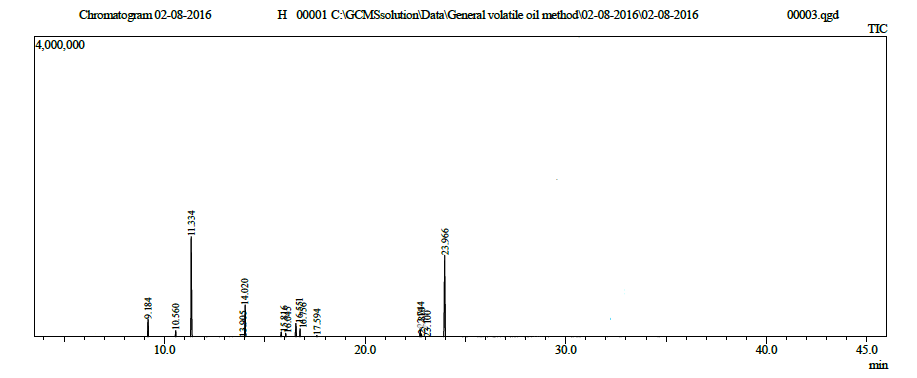


Fig S2: GC/MS chromatogram of Myrrh essential oil

Supplement: Supplementary file 1 — Additional file 1: Figure S1. GC/MS chromatogram of Myrrh hexane extract. Figure S2. GC/MS chromatogram of Myrrh essential oil. [file 13568_2020_958_MOESM1_ESM.docx]
